# Volumetric analysis of spontaneous bone healing after jaw cyst enucleation

**DOI:** 10.1038/s41598-022-16921-w

**Published:** 2022-09-02

**Authors:** Jeong-Kui Ku, Michael Han, Atapol Yongvikul, Jong-Ki Huh, Jae-Young Kim

**Affiliations:** 1 Department of Oral and Maxillofacial Surgery, Gangnam Severance Hospital, Yonsei University Health Service, Seoul, Republic of Korea; 2 Department of Oral and Maxillofacial Surgery, University of Illinois Chicago, Illinois, USA; 3 Department of Oral and Maxillofacial Surgery, Gangnam Severance Hospital, Yonsei University College of Dentistry, 211 Eonju-ro, Gangnam-gu, Seoul, 06273 Republic of Korea; 4 Present Address: Department of Oral and Maxillofacial Surgery, Masterpiece Hospital, Bangkok, Thailand

**Keywords:** Cysts, Maxillofacial surgery, Oral surgery

## Abstract

The purpose of this study is to evaluate the degree of spontaneous bone healing after cyst enucleation as well as its contributing factors. Pre- and post-operative computed tomography (CT) scans of consecutive patients who had undergone jaw cyst enucleation were retrospectively analyzed. The outcome variable was healing ratio, which was calculated using the volume of the cyst before surgery and the volume of the defect in the bone after surgery. Predictor variables including duration of observation, pre-operative cyst size, age, gender, and involved jaw were analyzed to determine their influence. Forty-four subjects (30 Male and 14 Female, average 40.7 ± 15.7 years) were included in this study. Healing ratio was significantly lower during the first year (33.5 ± 32.8%) compared to the second (74.5 ± 24.2%) and subsequent years (74.2 ± 17.8%). In 35 patients who had follow-ups of over 1 year, the healing ratio was not affected by the pre-operative cyst size and upper/lower jaw except gender (*p* = 0.037, female > male) and age (*p* = 0.021, younger than 30 years > 30 years and older). The residual defect was significant larger in cysts 3 cc or larger (1.64 ± 1.54 cc) compared to smaller cysts (0.43 ± 0.42 cc, p = 0.006). The residual defect volume of large cysts was similar to those of the pre-operative volume of small cysts (1.47 ± 0.72 cc). In conclusion, spontaneous bone healing ratio of post-enucleation defects was about 73.5% after 12 months. Large cysts (> 3 cc) had larger defect, comparable to the volume of small cysts, but with altered contour. Additional treatment such as a bone graft may be considered especially in large cysts.

## Introduction

Cysts of the jaw are mostly well-circumscribed oval-shaped radiolucent lesions with various characteristics in size and origin^1^. Complete enucleation is a well-established management option, with relatively infrequent postoperative complications such as infection and incomplete bone healing^2,3^. Studies evaluating post-enucleation defects have mainly used 2-dimentional (2D) imaging such as panoramic radiographs. Rubio and Mombru reported in a randomized clinical study that total spontaneous bone regeneration was 88.5% at least 6 months after the enucleation without bone grafts, and that the healing rate was not affected by the cyst type^4^. Older individuals (more than 30 years) have been shown to have less bone healing compared to younger ones^2,5^. With regards to the influence of the affected jaw, the data has been equivocal while some research report unfavorable bone regeneration after enucleation of maxillary cysts^6–8^.

It is well-known that postoperative bone healing is affected by the size of the cyst defect and duration of observation after the surgery^9,10^. Depending on the size of the cyst, the prognosis for small cysts can be satisfactory with minimal morbidity. Yim and Lee observed about 97% regeneration of bone density 12 months postoperatively in small defects of 2–3 cm^11^. In a systematic review, Nyimi et al. concluded that small defects of less than 4 cm can be regenerated to sufficient bone density within 24 months after enucleation^12^. However, treatment of large cysts has been challenging because large cysts have been shown to have a greater postoperative risks such as pathologic fracture, limited opening, and insufficient bone healing^13,14^. Although pathologic fracture is rare after enucleation, there was a fracture rate of 3.1% in a study on 160 patients presenting with cysts of the mandibular angle with a mean size of 31.5 mm, with most being males^8,15^.

In 2021, Vitale et al. evaluated postoperative healing in 15 mandibular radicular cyst enucleations using 3-dimensional (3D) volumetric analysis, and found near complete bone healing irrespective of the pre-operative defect size (1.3–19.5 cc) although there was alteration of the mandibular profile when there was significant loss of cortical bone^16^. However, in terms of spontaneous bone regeneration with cyst enucleation, there is still no consensus considering several factors such as pre-operative cyst size, age, gender, and involved jaw. This study aims to evaluate the spontaneous bone healing after the cyst enucleation according using a 3D volumetric protocol analyzing several predictor variables.

## Material and methods

The institutional review board of Yonsei University Gangnam Severance Hospital approved this retrospective study (IRB No. 3-2021-0324), which was conducted according to the principles of the Declaration of Helsinki for research on humans.

Consecutive patients treated with enucleation by the Department of Oral and Maxillofacial Surgery at Gangnam Severance Hospital from March 2011 to March 2021 for developmental odontogenic (such as dentigerous cyst or odontogenic keratocyst) or inflammatory cysts (such as radicular cyst) in accordance with the World Health Organization’s updated classification were reviewed^17^. The inclusion criteria were as follows: (1) Cyst enucleation under general anesthesia with peripheral ostectomy; (2) healthy status or well-controlled systemic diseases; (3) pre-operative CT taken within one month of cyst enucleation; and (4) post-operative CT taken at least 6 months after enucleation. The exclusion criteria were as follows: (1) age under 19 years; (2) uncontrolled systemic diseases and oral hygiene management; (3) decompression before the cyst enucleation; (4) bone graft after the surgery; (5) recurrence of the cyst; (6) post-operative complications such as wound dehiscence or infection; and (7) unavailable or insufficient imaging.

The MDCTs were taken with subjects positioned according to the manufacturer’s instructions (Siemens definition AS+, Siemens, Germany), with their teeth in centric occlusion. MDCT formats were obtained using a scanner with the following setting: 1-mm thickness, 7-s scan time, 120 kV, and 90 mAs. CBCT images were also acquired using a scanner (PaX-i3D, Vatech Co., Gyeonggi-do, Republic of Korea) using one setting: 0.3-mm, 24 s, 106 kV, and 65 mAs.

**Figure 1:**
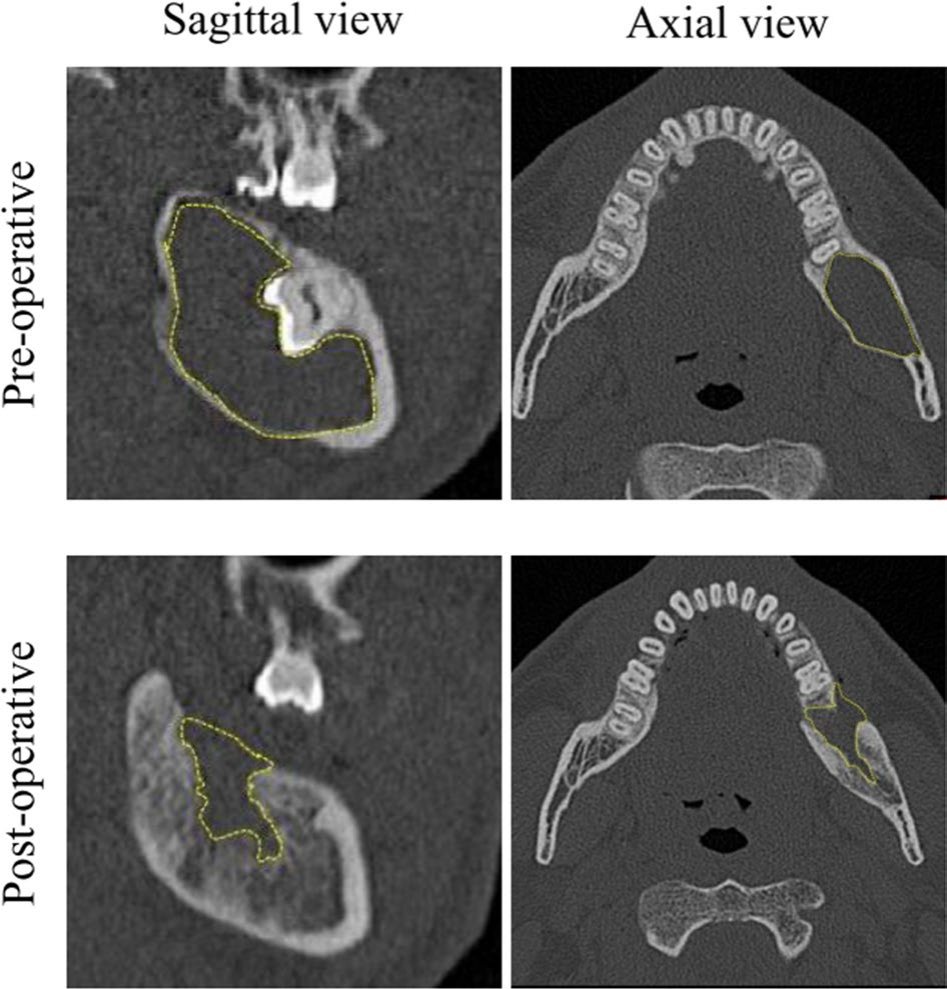
3D measurement protocol by using computed tomography. After a ROI was delineated in one section, the border of the defects (yellow dotted line) was automatically generated for each slice on the axial and sagittal views, respectively. The defect volume was calculated through 3D reconstruction.

Materialise’s interactive medical image control system (MIMICS V21.0, Materialise, Leuven, Belgium) was used to measure the volume^18^. The CT images were converted into the Digital Imaging and Communications in Medicine (DICOM) files, and they were imported into MIMICS. The specific threshold value (370-3071 Hounsfield units for bone demonstration) was reconstructed for measurement^19,20^. Once a region of interest (ROI) was automatically delineated in each sample, one examiner (AY) confirmed the border of the pre-operative cyst or residual defects on the axial and sagittal views, respectively. The defect volume was automatically generated by 3D reconstruction (Fig. 1) . Regarding pre- and post-operative defect volume, the healing ratio—which was the primary outcome variable—was calculated as follows:

$$\mathrm{Healing \, ratio }(\mathrm{\%}) = \frac{Preoperative \, cyst \, volume \, (cc) - Postopeartive \, infrabony \, defect \, volume \, (cc)}{Preoperative \, cyst \, volume \, (cc)}\times 100$$

The measurements were performed twice with a 3-week washout period. For repeated measurements with an intraclass correlation coefficient of higher than 0.999, we used the averaged values between two measurements for the defect volume.

### Statistical analysis

The parametric assumption of the data was analyzed by using the Kolmogorov–Smirnov test. Simple regression analysis was performed to analyze the relationship between the pre-operative defect volume and healing ratio after cyst enucleation. The duration of observation was classified into the three groups: (1) between 6 months and 1 year; (2) between 1 and 2 years; and (3) greater than 2 years. Kruskal–Wallis test was used to analyze the healing ratio with respect to the classified duration of observation, and post hoc analysis was performed by Bonferroni correction. The primary outcome variable was healing ratio. The healing ratio was analyzed using Mann–Whitney U test according to the independent variables, which were pre-operative cyst volume (less than or equal to vs. more than 3 cc), age (less than vs. equal to or more than 30 years), gender, and involved jaw (upper vs. lower jaw). Subjects with follow-up and post-operative imaging at least 1 year after cyst removal were analyzed separately to assess the influence of longer duration of observation. SPSS 25.0 statistical software (IBM Corp; Armonk, NY) was used for statistical analysis. A *p* value of less than 0.05 was considered statistically significant.

### Ethical approval

The study was approved by the institutional review board of Yonsei University Gangnam Severance Hospital approved this retrospective study (IRB No. 3-2021-0324), and waiver of written informed consent for this retrospective study.

## Results

Forty-four subjects (30 males and 14 females, mean age 40.7 ± 15.7 years) were included in this study. Overall, the mean volume of the cyst and residual defects were 3.98 ± 3.57 and 1.17 ± 1.21 cc, respectively, and the healing ratio was 66.0 ± 28.3% at 2.5 ± 1.9 year postoperative (Table 1). There was no significant difference in the healing ratio based on pre-operative cyst size, gender, age, and involved jaw. (Table 1).

**Table 1:** Volumetric analysis for spontaneous bone healing after cyst enucleation.

Variables	Age (years)	Pre-operative volume (cc)	Duration of observation (year)	Healing Ratio (%)
Total (n = 44)	40.7 ± 15.7	3.98 ± 3.57	2.5 ± 1.9	66.0 ± 28.3
**Pre-operative cyst volume**				
< 3 cc (n = 22)	41.6 ± 15.1	1.47 ± 0.73	2.1 ± 2.9	63.4 ± 28.2
≥ 3 cc (n = 22)	36.7 ± 16.5	6.36 ± 3.72	2.9 ± 2.1	66.5 ± 29.6
p value*	0.290	< 0.001^**†**^	0.226	0.254
**Gender**				
Male (n = 30)	40.7 ± 15.7	4.44 ± 3.80	2.4 ± 2.0	60.8 ± 29.0
Female (n = 14)	36.7 ± 17.6	2.98 ± 2.88	2.7 ± 1.8	77.1 ± 24.1
p value*	0.319	0.096	0.036^**†**^	0.412
**Age**				
< 30 years (n = 13)	21.6 ± 4.74	4.08 ± 3.35	2.7 ± 2.2	77.5 ± 25.5
≥ 30 years (n = 31)	47.0 ± 13.14	3.93 ± 3.71	2.4 ± 1.81	61.2 ± 28.4
p value*	< 0.001^**†**^	0.671	0.015^**†**^	0.827
**Jaw**				
Mandible (n = 24)	35.5 ± 15.7	4.11 ± 3.87	2.7 ± 2.3	69.3 ± 30.6
Maxilla (n = 20)	44.3 ± 16.0	3.81 ± 3.26	2.3 ± 1.4	62.0 ± 25.5
p value*	0.071	0.741	0.083	0.981

*Mann–Whitney U test.

^**†**^*p* value < 0.05.

Regarding the duration of observation, age and pre-operative cyst volume were similarly distributed. However, the healing ratio was significantly lower during the first year (33.5 ± 32.8%) compared to the second year (74.5 ± 24.2%) and subsequent years (74.2 ± 17.8%). (Table 2).

**Table 2:** Volumetric analysis of spontaneous bone healing after cyst enucleation based on duration of observation.

Duration of observation	Age (years)	Pre-operative volume (cc)	Healing ratio (%)
≤ 1 year (n = 9)	40.3 ± 14.7	2.86 ± 2.06	33.5 ± 32.8^a^
1–2 years (n = 14)	33.9 ± 15.4	3.67 ± 4.42	74.5 ± 24.2^b^
> 2 years (n = 21)	42.8 ± 17.1	4.66 ± 3.44	74.2 ± 17.8^c^
p value*	0.330	0.283	0.002
Post-hoc**			a vs. b: 0.006 a vs. c: 0.003 b vs. c: > 0.999

*Kruskal–Wallis.

**Mann–Whitney U test with Bonferroni’s correction.

There were 35 subjects (23 male and 12 female, mean age 40.7 ± 16.2 years) with follow-up and post-operative imaging at least 1 year after cyst removal. In this subgroup, the mean pre-operative and residual defects were 4.20 ± 3.92 and 1.05 ± 1.28 cc, respectively, and the healing ratio was 73.5 ± 20.6% after 3.0 ± 1.9 year postoperative (Table 3).

**Table 3:** Volumetric analysis for spontaneous bone healing for long-term follow-up group (≥ 1 year).

Variables	Age (years)	Pre-operative volume (cc)	Duration of observation (year)	Healing ratio (%)
Total (n = 35)	40.7 ± 16.2	4.20 ± 3.92	3.0 ± 1.9	73.5 ± 20.6
**Pre-operative cyst volume**				
Under 3 cc (n = 16)	41.9 ± 17.3	1.47 ± 0.72	2.6 ± 1.7	73.7 ± 18.0
Over 3 cc (n = 19)	39.6 ± 15.5	6.77 ± 3.97	3.4 ± 2.0	73.3 ± 23.3
p value*	0.367	< 0.001^†^	0.422	0.367
**Gender**				
Male (n = 23)	41.8 ± 15.0	4.80 ± 4.28	2.6 ± 0.5	68.2 ± 22.7
Female (n = 12)	38.6 ± 18.8	3.00 ± 2.90	2.7 ± 0.5	84.0 ± 9.4
p value*	0.344	0.248	0.572	0.037^†^
**Age**				
Under 30 (n = 12)	23.2 ± 4.1	3.93 ± 3.73	3.0 ± 2.4	82.8 ± 13.9
Over 30 (n = 23)	48.3 ± 13.2	4.32 ± 4.08	3.0 ± 1.7	69.4 ± 21.9
p value*	< 0.001^†^	0.959	0.278	0.021^†^
**Jaw**				
Mandible (n = 21)	36.0 ± 17.2	4.43 ± 4.18	2.6 ± 0.5	78.6 ± 22.3
Maxilla (n = 14)	43.1 ± 15.9	4.07 ± 3.50	2.6 ± 0.5	69.3 ± 16.8
p value*	0.061	0.803	0.198	0.630

*Mann–Whitney U test.

^†^*p* value < 0.05.

**Figure 2:**
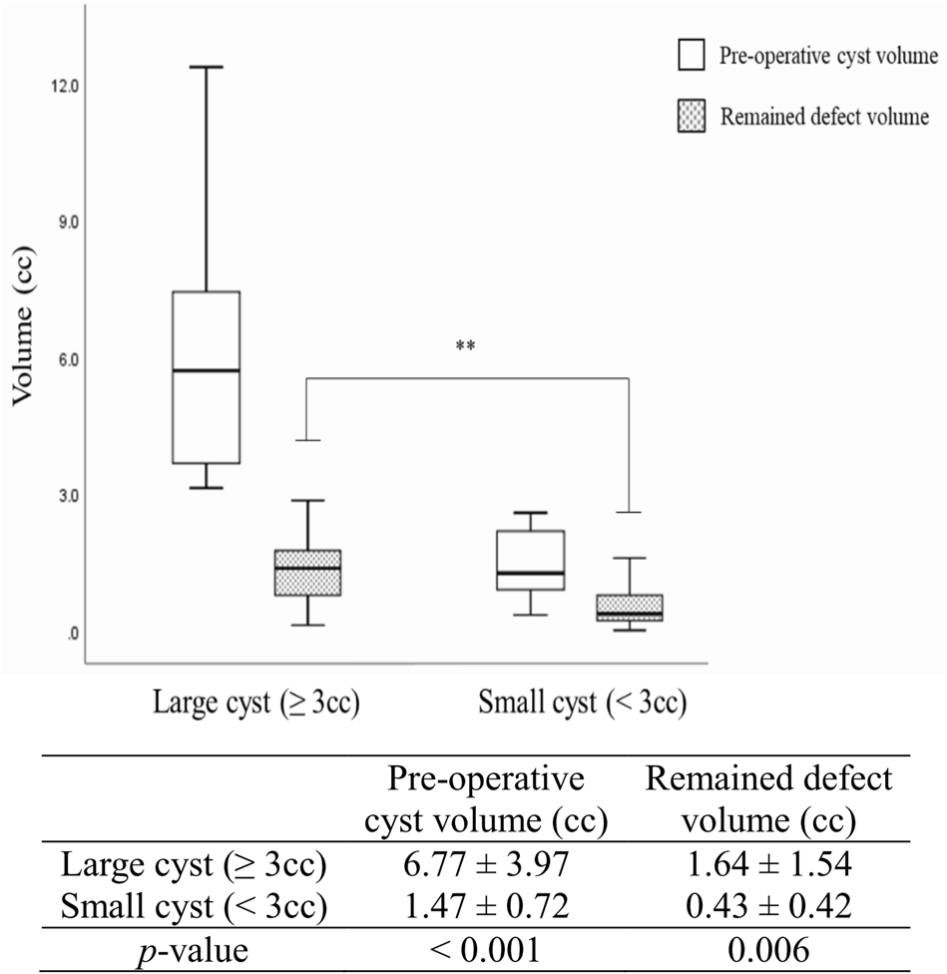
Comparison of pre-operative cyst and remained defect size between the large and small cysts (over one year). The residual defect size was significantly different between the large and small cysts (p < 0.001). The residual defect volume of large cysts was similar to the pre-operative cyst volume of the small cyst group.

Healing ratio showed no significant difference based on pre-operative cyst size and involved jaw. However, gender and age significantly affected the healing ratio (Table 3). Female subjects had significantly higher bone healing ratio (84.0 ± 9.4%) than males (68.2 ± 22.7%, p = 0.037, Table 3). Younger patients (< 30 years) had higher bone healing ratio (82.8 ± 13.9%) than older patients (69.4 ± 21.9, p = 0.021, Table 3). There was no significant difference between large and small cysts in the healing ratio, with large cysts having larger defects (1.64 ± 1.54 cc) than in small cysts (0.43 ± 0.42 cc, p = 0.006). In addition, the residual defect volume of the large cyst group was similar to the pre-operative volume of the small cyst group (1.47 ± 0.72 cc) (Fig. 2).

## Discussion

Cysts of the jaws are common, and can cause expansion and destruction of maxillofacial bones. While many previous investigations demonstrated enucleation to be a cost-effective and safe method resulting in good bone healing^2,3^, the healing of post-enucleation defects has been evaluated mostly by bone density and 2D measurements^2,4–8^. In this 3D study, enucleation of jaw cysts showed similar bone regeneration regardless of the pre-operative cyst size, age, and involved jaw. Although the bone regeneration rate was only 33.5% in the first year, the healing ratio reached about 75% after the first postoperative year without complications. These results are consistent with those reported by Chacko et al., where complete bone healing was achieved in almost all patients 12 months after enucleation^21^.

With regards to large defects (> 4 cm) in the mandible, some authors have reported postoperative bone density increases by 27–37% and 46–48% at 6 and 12 months, respectively^2,3^, but with sufficient increase after 24 months despite the large size. In a recent study by Vitale et al., the residual intrabony defect after mandibular radicular cyst enucleation was calculated 3-dimentionally, and the intrabony defects were filled 99.72% at 12 months after surgery^16^. According to this study, however, the greatest residual volumes were found at both 6 months and 12 months for larger cysts. It is worthy of note that while this study described a fully 3D measurement protocol, but it was not possible to confirm whether the method was automatically calculated. In this study, the boundary was marked by manually checking images from each sample at 1 mm section intervals in consideration of the altered contour due to incomplete regeneration of cortical bone. To sensitively show the incomplete regeneration, this study emphasized the bone contour with considering the normal cortex on the non-affected site, and showed that the bone regeneration was not completed during 12 months.

Bone healing is affected by the location of the defects with the surrounded bone and periosteum, which have healing capacity for spontaneous bone repair^22^. Intraosseous cysts, especially in the mandible, are frequently completely surrounded by sound bone walls except for where surgical access is made. However, large mandibular or maxillary cysts usually leave extraosseous cystic defects due to perforation through the cortex, and sometimes into the maxillary sinus. Although several studies using 2D radiography measuring bone density reported significant roles of the size and location of the cyst on postoperative healing^2,3,6–8^, our study using 3D analysis did not. The residual defect was generally located in the center, suggesting a centripetal intraosseous healing path with newly generated cortical bone^16^. Such patterns may appear as normal bone density on 2D radiography even when bone healing is incomplete (Fig. 1), which could explain the different findings in other studies using 2D analysis.

One limitation of this study is not accounting for bone loss from surgical access, as well as from simultaneous tooth extraction. Also, the sample size of subjects with long-term follow-up (n = 35) with a somewhat heterogeneous distribution based on the predictor variables warrant careful interpretation of the data. Within these limitations, this study showed incomplete spontaneous bone healing of cyst enucleation defects without bone grafting, especially in men or in cysts larger than 3 cc. The effectiveness of bone grafting for unfavorable cyst defects remains to be fully revealed by well-designed clinical research in the future.

## Conclusion

The spontaneous bone healing ratio of defects was 73.5% after 12 months after enucleation. Large cysts of greater than 3 cc had larger defects which were similar in size to the volume of small cysts. Additional treatment such as a bone grafting may be considered especially in large cysts.
